# Cervical cancer screening among US women with pelvic organ prolapse

**DOI:** 10.21203/rs.3.rs-10537590/v1

**Published:** 2026-09-11

**Authors:** Carla L Da Goia Pinto, Laura Crespo-Albiac, Tara Master Hunter, Paul Lin, Madeline Moureau, Neil Kamdar, Courtney Streur, Diane M Harper

**Affiliations:** University of Illinois Chicago; University of Michigan; University of Michigan; University of Michigan; University of Michigan; University of Michigan; University of Michigan; University of Michigan

## Abstract

**Introduction:**

Cervical cancer occurs in women with pelvic organ prolapse (POP), but the prevalence of cervical cancer screening (CCS) in this population is unknown. We hypothesize that age, race, ethnicity, and college attendance will impact CCS for women with POP.

**Methods:**

In this cross-sectional study, we extracted CCS laboratory codes from an electronic healthcare database, Truveta, between 2017 and 2022 for women aged 21-65 who were without a hysterectomy or prior gynecologic cancer. We extracted POP data based on medical codes.

**Results:**

Among 2,265,395 eligible women, we found a 4.4/1000 prevalence of POP among women aged 21-65 years who were eligible for CCS. Women with POP are significantly older (50-65 years old: 63.8% vs 35.2%, p<0.001), White (84.5% vs 73.6%, p<0.001), and have less college education (43.2% vs 59.9%, p<0.001) than women without POP. CCS among women with POP was greater than in women without POP (72.6% vs 70.8%, p<0.001). In analyses adjusted for age, race, ethnicity, and college education, they were 56% more likely to have CCS (aOR=1.56 (95% CI: 1.44, 1.77), p<0.001) than women without POP. In addition, Asian, Black, Hispanic, and White women with POP had greater adjusted odds of CCS than their respective groups without POP.

**Conclusion:**

Women with POP have greater CCS rates than those without. CCS rates for women with POP who are 50 and older, the age group at highest risk for cervical cancer, are still below the Healthy People 2030 (HP2030) goals for cervical cancer screening.

## Introduction

Cervical cancer screening (CCS) is a powerful secondary prevention tool for cervical cancer elimination. The Healthy People 2030 (HP2030) aims for 79.2% of CCS-eligible women to receive their CCS, where the most recent data indicate an overall baseline of 73.9% [1], a rate that has not changed in thirty years. The median age of cervical cancer is 50 years, with 40% of cervical cancers presenting in women aged 55 and older [2]. Yet, the CCS rate for those 50 and older is the lowest of all women screened [3]. Pelvic organ prolapse (POP) presents at an average age of 55 years, intersecting with the age of highest cervical cancer risk [4].

Cervical cancer occurs in women with pelvic organ prolapse (POP) [5], and women undergoing POP surgical treatment with cervix removal have a 6.9/1000 prevalence of previously unidentified cervical intraepithelial neoplasia grade 2 or worse (CIN2+) [6]. Pelvic organ prolapse (POP) includes urinary anterior vaginal wall prolapse (40%), rectal posterior vaginal wall prolapse (15%), and cervico-uterine prolapse (15%), or multiple organ prolapses (30%) [7].

The prevalence of POP is reported to increase with increasing age, with increasing parity, and among women who have had a hysterectomy [8]. POP is also noted to be more common among women with less education compared to those with some college, and among those who delivered a child by vaginal delivery [8]. However, racial and ethnic differences in population-based POP research are limited [9], leading to knowledge deficits in health equity outcomes for the intersecting effects of POP on CCS. We aim to describe the prevalence of women with POP who undergo CCS (2017–2022) and describe the covariates of age, race, ethnicity, and education impacting their rates of CCS.

### Methods –

We used a cross-sectional study design, with exempt IRB status from the University of Michigan ethics review board, using 2016 as a lookback year to identify women who were 21–65 years old during the 2017–2022 study period who were eligible for CCS. We used a national electronic health records database (Truveta) with individual-level demographic information, linked to LexisNexis for educational attainment, representing over 130 million people across the United States [Appendix A].

All women had to have at least two unique health maintenance visits in separate years during the study period, as a proxy for continuous 2017–2022 health care utilization within the Truveta data source; we did not restrict the taxonomy of the person who recorded the HMV in the electronic health record. CCS was defined by a laboratory code, not the visit type.

United States Preventive Services Task Force (USPSTF) guidelines for CCS did not change during the study period: recommending every 3 years for those 21–29 and every 5 years for those 30–65 years [3, 4]. Women were excluded if they died during the study period, had a hysterectomy, except for those being followed after treatment for high-grade cervical intraepithelial neoplasia [Appendices B-E]. POP was defined by its International Classification of Diseases, Ninth or Tenth revision, Clinical Modification (ICD-9) medical codes (618.0-618.9) and ICD-10 medical codes (N81.0-9). Age, race, and ethnicity are self-reported in the health record. Other race refers to multirace or races not herein named.

## Results

We identified 2,255,487 women without POP and 9,908 women with POP. Figure 1 displays both the populations of women with and without POP (left, log-axis, columns) and the CCS for each of the two populations (right axis, dots). We used the log axis for population counts to display the smaller race categories not previously presented in the literature. Table 1 focuses on the population distributions. We show significantly greater orders of magnitude more population without POP than with POP overall, between all age groups, all races, ethnicities, and college attendance (p < 0.001).

Population. Overall, the prevalence of POP was 4.4/1000 among women aged 21–65 who were CCS-eligible. Women with POP were considerably older than those without POP (50–65 years: 63.8% vs 35.2%, p < 0.001, respectively). White women made up a greater proportion of the population with POP than without (84.5% vs 73.6%, p < 0.001); whereas, Asian (6.3% vs 3.6%, p < 0.001), Black (15.1% vs 7.9%, p < 0.001), Native Hawaiian/Pacific Islander(NHPI) (0.4% vs 0.3%, p = 0.01), and Other (4.1% vs 3.3%, p < 0.001) races, as well as Hispanic women (8.5% vs 6.4%, p < 0.001), had significantly greater proportions without POP than with POP.

Cervical Cancer Screening. Table 2 shows that overall, women with POP had greater CCS rates than women without POP (72.6% vs 70.8%, p = 0.0001). For each descriptor (age, race, ethnicity, or education), women with POP had greater CCS rates than women without POP.

Table 3 presents the odds of CCS by POP adjusted for age, race, ethnicity, and education. POP was independently associated with increased odds of CCS (aOR = 1.60 (95% CI: 1.44, 1.77), p < 0.001). This association held across the age groups, with the exception of women aged 21–29. Moreover, Asian, Black, White, and Hispanic women with POP had significantly greater adjusted odds of CCS compared to those without POP (aOR = 1.60 (1.04, 2.46, p = 0.02; aOR = 1.71 (1.28, 2.27), p < 0.001; aOR = 1.58 (1.35, 1.86), p < 0.001, respectively). Finally, education was associated with greater adjusted odds of CCS in both POP groups (POP: aOR = 1.64 (1.42, 1.90), p < 0.001; no POP: aOR = 1.53 (1.31, 1.79), p < 0.001).

Table 4 displays the adjusted odds of CCS for the population of women with POP. Within this population, women 40 and older have significantly lower odds of CCS than women 39 and younger by every pairwise comparison. Specifically, women aged 50–65 years have a 72% lower chance of CCS than a 21–29 year old (aOR = 0.28, 95% CI: 0.12, 0.62, p < 0.001). No other covariates were associated with CCS in the POP population.

## Discussion

Our analysis provides new evidence about CCS among women with POP previously missing from the literature [10, 11, 12]. First, we built on Kenne’s work [13], wherein age was heretofore associated with increased odds of POP as age increased, and Asian and Black women were less likely to have POP than White women. Our work supports the increase in the prevalence of POP as women age, and that the majority of women with POP are White. We show through epidemiologic analysis that the lack of college education is associated with greater POP, a finding also found through multivariable Mendelian randomization [14]. Finally, our prevalence of POP was 4.4/1000 among women aged 21–65 who were CCS-eligible, lower than the general population because we omitted the oldest women who have the most prolapse [8].

CCS has not reached the Healthy People decennial goal for the past three decades [15]. Prior work established that women with POP have previously undetected CIN 2 + lesions at the time of prolapse surgery [5, 16], emphasizing the importance of CCS in this population. In our study, the CCS rates among women with POP aged 21–29 and 30–39 years exceeded the HP2030 goal of 79.2% [17], as did the rates among Asian and NHPI women with POP, regardless of age. We hypothesize that young women with POP are more aware of their prolapse, as this is the reproductive age range during which they seek health care, triggering the CCS. There are no data on whether women with POP get CCS more often than recommended.

In contrast, women aged 50 and older, who account for nearly half of cervical cancers, remain underscreened regardless of POP. While higher education is associated with greater odds of CCS among women with POP, its impact is insufficient to meet the HP2030 goal for older adult women, underscoring the need for targeted clinical strategies among older women, most likely postmenopausal. Other work has proven that postmenopausal women prefer CCS to be done with self-sampling, providing a possible improvement in health care in the future [18].

## Conclusion

While POP appears to be strongly associated with CCS receipt, women over 50 are still underserved and deserve improved health care access.

## Supplementary Material

This is a list of supplementary files associated with this preprint. Click to download.

- Appendix.docx

## Figures and Tables

**Figure 1:**
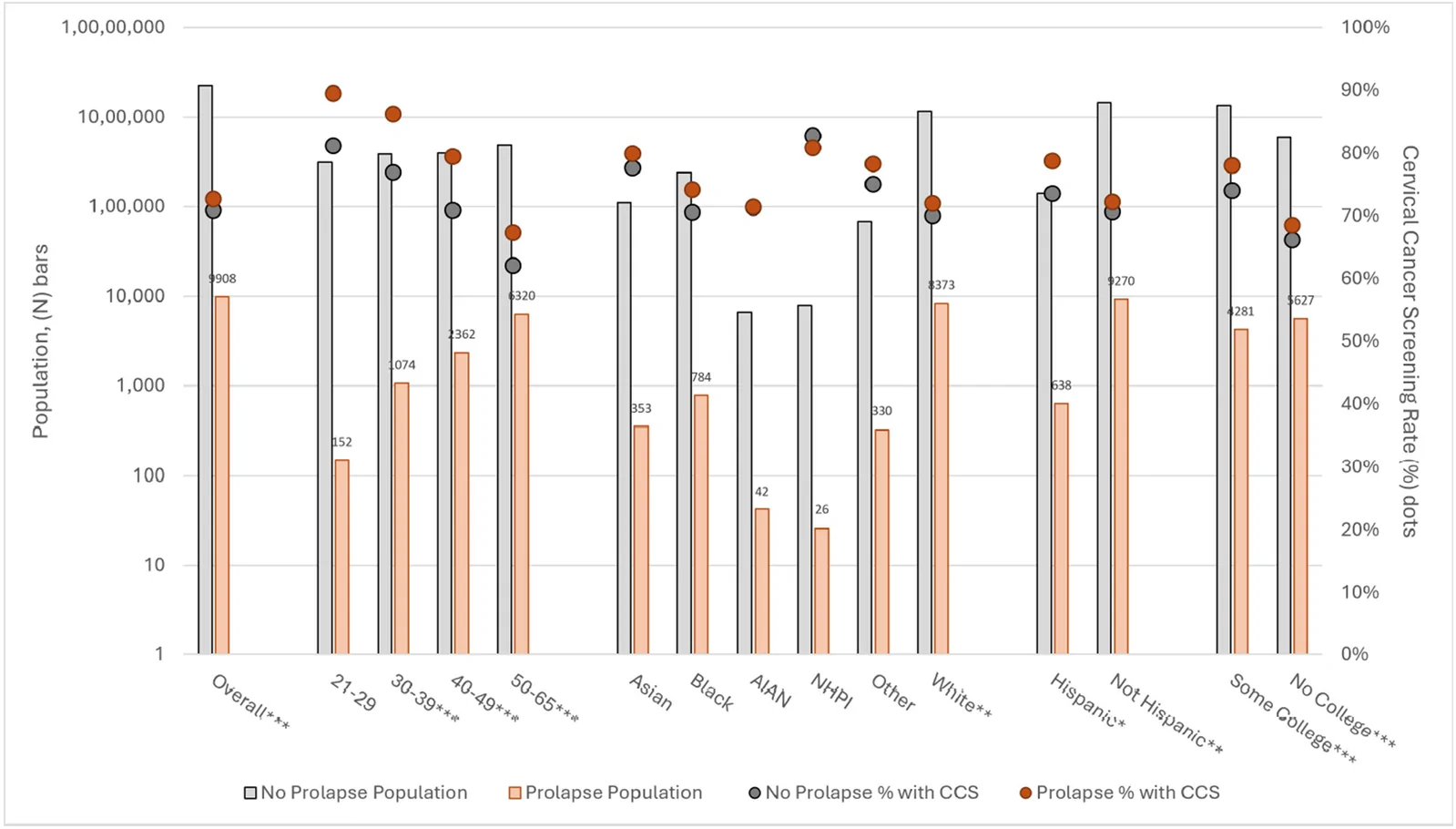
The proportion of women with pelvic organ prolapse (POP) and cervical cancer screening (CCS), 2017-2022. US population-based distribution of women with and without pelvic organ prolapse (POP) (left axis, log scale) and their respective cervical cancer screening (CCS) rates (right axis, percentage) by four covariates: Age, Race, Ethnicity, and Education Women 21-65 years old who are eligible for cervical cancer screening without hysterectomy or other cervical/gynecologic cancers. AIAN means American Indian/Alaska Native. NHPI means Native Hawaiian/Pacific Islander. Blue line is the Healthy People 2030 (HP2030) cervical cancer screening (CCS) goal of 79.2%.

**Table 1:** Population descriptors of cervical cancer screening (CCS) eligible women with pelvic organ prolapse (POP)

		Count (%)			P-value
		All	No POP	POP	
**Overall**		2,265,395 (100%)	2,255,487 (100%)	9,908 (100%)	--
**Age**					
	*Mean (SD)*	43.0 (11.7)	42.9 (11.7)	50.9 (8.4)	**< 0.001**
	*Median (Q1-Q3)*	44.0 (33.0–53.0)	44.0 (33.0–53.0)	53.0 (46.0–58.0)	**< 0.001**
**Age Groups**					
	*21–29*	386762 (17.1%)	386610 (17.1%)	152 (1.5%)	**< 0.001**
	*30–39*	510640 (22.5%)	509566 (22.6%)	1074 (10.8%)	
	*40–49*	567024 (25.0%)	564662 (25.0%)	2362 (23.8%)	
	*50–65*	800969 (35.4%)	794649 (35.2%)	6320 (63.8%)	
**Race**					
	*Asian*	143161 (6.3%)	142808 (6.3%)	353 (3.6%)	**< 0.001**
	*Black*	342313 (15.1%)	341529 (15.1%)	784 (7.9%)	
	*AIAN*	9352 (0.4%)	9310 (0.4%)	42 (0.4%)	
	*NHPI*	9652 (0.4%)	9599 (0.4%)	26 (0.4%)	
	*Other Race*	91992 (4.1%)	91662 (4.1%)	330 (3.3%)	
	*White*	1668952 (73.7%)	1660579 (73.6%)	8373 (84.5%)	
**Ethnicity**					
	*Hispanic*	191900 (8.5%)	191262 (8.5%)	638 (6.4%)	**< 0.001**
	*Non-Hispanic*	2073495 (91.5%)	2064225 (91.5%)	9270 (93.6%)	
**Education**					
	*Some College*	1354291 (59.8%)	1350010 (59.9%)	4281 (43.2%)	**< 0.001**
	*No College*	911104 (40.2%)	905477 (40.1%)	5627 (56.8%)	

Statistical tests for continuous variables: T-test/Wilcoxon Test

Statistical tests for categorical variables: Chi-square/Fisher’s Exact Test.

Bold is significant.

AIAN means American Indian/Alaska Native

NHPI means Native Hawaiian/Pacific Islander

Cancer screening eligible women are 21–65 years without hysterectomy or cervical/gynecological cancers

**Table 2:** Cervical cancer screening (CCS) among women according to pelvic organ prolapse (POP)

CCS Rate	Cervical Cancer Screening Rate			P-Value
	All	No POP	POP	
**Overall**	1604884/2265395 (70.8%)	1597692/2255487(70.8%)	7192/9908 (72.6%)	**< 0.001**
**Age Groups**				**< 0.001**
*21–29*	313509/386762 (81.1%)	313373/386610 (81.1%)	136/152 (89.5%)	0.183
*30–39*	392738/510640 (76.9%)	391812/509566 (76.9%)	926/1074 (86.2%)	**< 0.001**
*40–49*	401397/567024 (70.8%)	399521/564662 (70.8%)	1876/2362 (79.4%)	**< 0.001**
*50–65*	497240/800969 (62.1%)	492986/794649 (62.0%)	4254/6320 (67.3%)	**< 0.001**
**Race**				0.776
*Asian*	111159/143161 (77.6%)	110877/142808 (77.6%)	282/353 (79.9%)	0.978
*Black*	6665/9352 (71.3%)	6635/9310 (71.3%)	30/42 (71.4%)	1.000
*AIAN*	7957/9625 (82.7%)	7936/9599 (82.7%)	21/26 (80.8%)	1.000
*NHPI*	241478/342313 (70.5%)	240897/341529 (70.5%)	581/784 (74.1%)	0.369
*Other Race*	83612/110969 (75.3%)	83303/110571 (75.3%)	309/398 (77.6%)	0.970
*White*	1168635/1668952 (70.0%)	1162615/1660579 (70.0%)	6020/8373 (71.9%)	**0.004**
**Ethnicity**				**0.040**
*Hispanic*	141127/191900 (73.5%)	140625/191262 (73.5%)	502/638 (78.7%)	**0.019**
*Non-Hispanic*	1463757/2073495 (70.6%)	1457067/2064225 (70.6%)	6690/9270 (72.2%)	**0.005**
**Education**				**0.024**
*Some College*	1002834/1354291 (74.0%)	999496/1350010 (74.0%)	3338/4281 (78.0%)	**< 0.001**
*No College*	602050/911104 (66.1%)	598196/905477 (66.1%)	3854/5627 (68.5%)	**< 0.001**

AIAN means American Indian/Alaska Native

NHPI means Native Hawaiian/Pacific Islander

Other means any other race not listed or multirace.

Cervical Cancer Screening (CCS) eligible women are 21–65 years without hysterectomy, or cervical/gynecological cancers

**Bold is** significant from Simple Logistic Regression with Firth correction, with post hoc Tukey Test applied for pairwise comparison.

**Table 3:** The odds of cervical cancer screening by pelvic organ prolapse (POP), adjusting for age, race, ethnicity, and education, 2017–2022

		Main	Reference	aOdds Ratio (95% CI)	Tukey P-value
*Prolapse (No Interaction)*		*Had Prolapse*	*No Prolapse*	**1.595 (1.441–1.765)**	**< .001**
*Age Group*	*21–29*	*Had Prolapse*	*No Prolapse*	1.901 (0.862–4.194)	0.212
	*30–39*	*Had Prolapse*	*No Prolapse*	**1.873 (1.428–2.458)**	**< .001**
	*40–49*	*Had Prolapse*	*No Prolapse*	**1.601 (1.366–1.876)**	**< .001**
	*50–65*	*Had Prolapse*	*No Prolapse*	**1.318 (1.182–1.470)**	**< .001**
*Race*	*AIAN/NHPI/Other*	*Had Prolapse*	*No Prolapse*	1.437 (0.942–2.193)	0.156
	*Asian*	*Had Prolapse*	*No Prolapse*	**1.602 (1.043–2.460)**	**0.02**
	*Black*	*Had Prolapse*	*No Prolapse*	**1.705 (1.283–2.266)**	**< .001**
	*White*	*Had Prolapse*	*No Prolapse*	**1.583 (1.350–1.857)**	**< .001**
*Ethnicity*	*Hispanic*	*Had Prolapse*	*No Prolapse*	**1.977 (1.470–2.660)**	**< .001**
	*Non-Hispanic*	*Had Prolapse*	*No Prolapse*	**1.563 (1.367–1.788)**	**< .001**
*Education*	*Some College*	*Had Prolapse*	*No Prolapse*	**1.641 (1.419–1.897)**	**< .001**
	*No College*	*Had Prolapse*	*No Prolapse*	**1.528 (1.305–1.790)**	**< .001**

Multivariate logistic regression with interaction terms with Firth correction. Tukey test applied for multiple pairwise comparisons.

AIAN means American Indian/Native American

NHPI means Native Hawaiian/Pacific Islander

Other means multirace or other race not named.

**Bold** indicates significance.

**Table 4.** The adjusted odds of cervical cancer screening among women with pelvic organ prolapse

	Main	Reference	aOdds Ratio (95%CI)	Tukey p-value
Age	*21-29*	*30-39*	1.31 (0.569-3.018)	0.977
	*40-49*	*30-39*	**0.624 (0.458-0.851)**	**<.001**
	*40-49*	*21-29*	0.477 (0.213-1.066)	0.097
	*50-65*	*30-39*	**0.363 (0.269-0.489)**	**<.001**
	*50-65*	*21-29*	**0.277 (0.124-0.618)**	**<.001**
	*50-65*	*40-49*	**0.582 (0.477-0.709)**	**<.001**
Race	*Asian*	*White*	1.432 (0.946-2.167)	0.146
	*Black*	*White*	1.078 (0.830-1.400)	0.989
	*AIAN/NHPI/Other Race*	*White*	1.086 (0.719-1.641)	0.999
	*Black*	*Asian*	0.753 (0.467-1.214)	0.618
	*AIAN/NHPI/Other Race*	*Asian*	0.758 (0.426-1.351)	0.833
	*AIAN/NHPI/Other Race*	*Black*	1.008 (0.624-1.628)	1.00
Ethnicity	*Hispanic*	*Non-Hispanic*	1.307 (0.984-1.735)	0.073
Education	*Some College*	*No College*	1.137 (0.986-1.312)	0.095

Multivariate logistic regression with interaction terms with Firth correction. Tukey test applied for multiple pairwise comparisons.

AIAN means American Indian/Native American

NHPI means Native Hawaiian/Pacific Islander

Other means multirace or other race

**Bold** indicates significance.

## Data Availability

Original data are not available. Programming language available by request to corresponding author.
